# Legal Notes for Institutional Workers

**Published:** 1915-04-03

**Authors:** 


### LEGAL NOTES FOR INSTITUTIONAL WORKERS.

### Insurance Policies.

There have been recently quite a crop of cases in which the judgment of the Court was more or less determined by medical considerations, and a brief account of one or two of these may be practically helpful to medical officers.

A life insurance policy provided that it did not "insure against death or disablement by accident directly or indirectly caused to any extent by medical or surgical treatment, or fighting, ballooning, racing, self-injury or suicide, or anything swallowed or administered or inhaled." The assured was found dead in a house of which he was sole occupant. His body was in the lavatory, where the gas was turned on, but not lighted. The insurance company repudiated liability on the ground that death was caused by "inhalation." The Court, however, found the company liable on the policy. This result was reached by application of the legal principle of construction of *ejusdem generis*, by which where in a contract or statute particulars are set forth and the clauses conclude with general words, the general words are not held to cover anything and everything indiscriminately, but only embrace such matters as are cognate in nature to the matters particularly mentioned. Accordingly in this case (*In re United London and Scottish Insurance Company*, 31 T.L.R. 203) the Court ruled that the word "inhaled" in the closing words of the clause quoted must be read *ejusdem generis* with the preceding words, and referred only to the inhalation for medicinal or other reasons of anything which the assured might believe to be harmless either *per se* or if taken in proper doses. "This construction," said Mr. Justice Astbury, "gives fair weight to the exception, is in harmony with the character of the excluded risks, and does no violence to the general character of the insurance." That is so, for were the company's view upheld, any accident from inhalation—*e.g.* a miner dying from the effects of poisonous gas—would be excluded, a consequence which is quite at variance with the main purport of the insurance contract itself.

### A New Field for Medical Witnesses.

We recently referred to two cases in which the difficult question of industrial diseases as affecting workmen's compensation was dealt with. We then explained fully how, if an industrial disease is of such a nature as to have been contracted by a gradual process, other employers who during the twelve months previous to the certificate of the certifying surgeon employed a workman suffering therefrom, are rendered liable to contribute towards the compensation which the Court may award to the workman. The last employer is the person against whom the claim for compensation has, in the first place, to be made by the workmen, but he in his turn can call on the other employers to contribute jointly with him. It would be a simple matter if, as the statute seems to assume would be the case, the only question were the apportionment of the sum payable in proportion to the respective period of employment. But a case has now arisen (*Barron* v. *Seaton Burn Coal Company*, 138 L.T.J. 381) which introduces fresh complications into a matter already sufficiently confused. Compensation was found due to a miner who suffered from miner's nystagmus. He had been employed in five different collieries within the year, and when it came to distributing the payment among these, one of the employers maintained that the disease was unlikely to have arisen in his employment, and that he is not liable in any proportion of the total weekly payment. The Court of Appeal have remitted to the County Court judge to try this issue. How he is to proceed, and what evidence he will call for, will cause that official no little perplexi[illegible] As one commentator on the case says, he must "do the best he can." In any event here is a new field for medical testimony and legal speculation.

